# Reciprocal Regulation of Annexin A2 and EGFR with Her-2 in Her-2 Negative and Herceptin-Resistant Breast Cancer

**DOI:** 10.1371/journal.pone.0044299

**Published:** 2012-09-05

**Authors:** Praveenkumar K. Shetty, Sanjay I. Thamake, Swati Biswas, Sonny L. Johansson, Jamboor K. Vishwanatha

**Affiliations:** 1 Department of Biochemistry, SDM College of Medical Sciences & Hospital, Dharwad, India; 2 Department of Biomedical Sciences, University of North Texas Health Science Center, Fort Worth, Texas, United States of America; 3 Department of Pathology, University of Nebraska Medical Center, Omaha, Nebraska, United States of America; 4 Department of Mathematical Sciences, The University of Texas at Dallas, Dallas, Texas, United States of America; University of Nebraska Medical Center, United States of America

## Abstract

Alternative survival pathways are commonly seen to be upregulated upon inhibition of receptor tyrosine kinases (RTK), including Her-2. It is established that treatment with Herceptin leads to selective overexpression and activation of epidermal growth factor receptor (EGFR) and Src which further contributes to oncogenesis in Herceptin resistant and triple negative breast cancer (TNBC) patients. Here, we show a co-regulated upregulation in the expression of Annexin A2 (AnxA2), a known substrate of Src and one of the regulators of EGFR receptor endocytosis, in Herceptin resistant and Her-2 negative breast cancer. Immunohistochemical expression analysis revealed a reciprocal regulation between Her-2 and AnxA2 in breast cancer clinical samples as well as in cell lines as confirmed by protein and RNA analysis. The siRNA and Herceptin mediated downregulation/inhibition of Her-2 in Her-2 amplified cells induced AnxA2 expression and membrane translocation. In this study we report a possible involvement of AnxA2 in maintaining constitutively activated EGFR downstream signaling intermediates and hence in cell proliferation, migration and viability. This effect was consistent in Herceptin resistant JIMT-1 cells as well as in Her-2 negative breast cancer. The siRNA mediated AnxA2 downregulation leads to increased apoptosis, decreased cell viability and migration. Our studies further indicate the role of AnxA2 in EGFR-Src membrane bound signaling complex and ligand induced activation of downstream signaling pathways. Targeting this AnxA2 dependent positive regulation of EGFR signaling cascade may be of therapeutic value in Her-2 negative breast cancer.

## Introduction

Her-2 (ErbB-2), Estrogen Receptor (ER) and Progesterone Receptor (PR) are the most commonly used biomarkers and therapeutic targets in breast cancer patients. However, these biomarkers are not expressed in 17–30% of women with breast cancer which limits the use of existing therapies [1]. Patients under hormone deprivation and Herceptin therapy, a most common therapeutic option, tend to acquire resistance to such therapies over time [2]. Whereas, the triple negative breast cancer (TNBC) phenotype, which lacks the presence of Her-2, ER and PR are even more aggressive and resistant [1], [3]. Therefore there is an urgent clinical need to identify new diagnostic as well as therapeutic markers for early diagnosis and treatment of such patients.

Herceptin, like other humanized receptor targeted monoclonal antibodies, inhibits the growth and progression in Her-2 positive breast tumors by blockade of downstream survival pathway(s) [4]–[8]. However, recent reports suggest that cells acquire resistance to the targeted therapies against receptor tyrosine kinases (RTKs) by several mechanisms [9], [10]. One of the most commonly seen mechanism is the activation of other receptor RTKs such as EGFR, IGFR and non-receptor tyrosine kinases such Src [10]. The overexpression of EGFR and Src in both Her-2 negative and TNBC cells contributes significantly to the tumor growth and progression [9], [11]–[17]. Considering the heterogeneity of cancer cells, it is predicted that not only these RTKs, but also other proteins which are required for normal functioning of these proteins are also upregulated in such cells [10], [17]. We found that Annexin A2 (AnxA2), a calcium dependent phospholipid binding protein, is inversely correlated with Her-2 expression. This observation holds true in case of Herceptin resistance, both in experimental and clinical situations.

AnxA2 is aberrantly expressed in various human cancers [18]–[24]. It is present as a monomer in the nucleus, but as a heterotetramer with p11 in the cytosol to bind to the inner and outer leaflets of the plasma membrane. The cytosolic AnxA2 is mobilized to the cell surface upon phosphorylation at the N-terminal Serine 25 (S25) and Tyrosine 23 (Y23), by different kinases such as PKC and Src as well as treatment with calcium ionophore or calcium inducing agents such as glutamate [25], [25,26]. The cell surface associated AnxA2 heterotetramer, is a receptor for both plasminogen and tissue type plasminogen activator (tPA) and acts as a catalytic center for the activation of plasminogen to plasmin [27], [28] which helps in invasion and metastasis of cancer cells [18], [27]. The membrane associated AnxA2 interacts with RTKs such as like insulin receptor (IR), insulin-like growth factor receptor (IGFR) and non-receptor tyrosine kinases such as focal adhesion kinase (FAK) and Src [29]–[33]. AnxA2 acts as a key scaffolding protein in anchoring and transportation of several proteins within plasma membrane as well as from cytosol to the plasma membrane, and contributes to cell signaling, angiogenesis and matrix degeneration [33]–[35]. Our recent data show that stimulation of AnxA2 by calcium ionophore or a phosphomimetic mutant of AnxA2 (Y23E) leads to its localization to the lipid raft component of the cell membrane, where it interact with different proteins and also leads to its own exosomal association [25]. Previous reports have shown that AnxA2 is involved in internalization and sorting of EGFR in early endosomes after ligand activation [34], [35]. It is also a known binding partner for Src kinase [33], which is present in EGFR signaling complex at the membrane [36], [37].

We report an inverse correlation between Her-2 and AnxA2 in breast cancer clinical samples and cell lines. This correlation was verified in Her-2 amplified cell lines and the functional relevance was validated in cell models of acquired resistance against Herceptin. We extended our studies to TNBC phenotype and validated the importance of AnxA2 in the EGFR receptor protein complex and consequent signaling cascade leading to cancer cell migration, proliferation and apoptosis. This is the first report implying the reciprocal regulation of Her-2 and AnxA2 and the role of AnxA2 in Her-2 negative breast cancer and EGFR signaling.

## Materials and Methods

### Cell Culture

Human breast cancer cell lines, HCC-70, HCC-1937, BT-549, HCC-1143, HCC-1187, HCC-38, HCC-1500, HCC-1569, MDA-MB-231, SK-BR-3, BT-474, MCF-7 and MCF-10A were obtained from ATCC and grown in respective medium as prescribed by the supplier. JIMT-1 cell line was procured from DSMZ (Brauenschweig, Germany) and grown in DMEM/F12 media with growth factors.

### Immunohistochemistry

Paraffin embedded tissue sections from the University of Nebraska Medical Center, Omaha, NE and St. Luke’s Regional Medical Center, Sioux City, IA., and tissue specimens from Accumax (ISU ABXIS Co., Ltd) and Biomax breast cancer tissue array (US Biomax, Inc.,) were used for immunohistochemical analysis performed as described previously [38]. AnxA2 mouse monoclonal antibody (BD Biosciences, CA. Catalog No. 610069) and Her-2 rabbit polyclonal antibody (Cell Signaling, MA. Catalog No. 2242) were used at 1∶100 dilutions. For the negative control, anti-rabbit and anti-mouse IgG whole molecule (Sigma–Aldrich, MO, Catalog No. I4506) was used at 1∶1000 dilution. Staining intensity of AnxA2 and Her-2 in neoplastic cells was graded on a scale of 0 (no staining) to 3+ (strong staining).

### Total Cell Extraction and Western Blotting

For the expression analysis in different breast cancer cell lines, total protein was extracted and quantitated as described previously [38]. Total protein was separated on 4–12% Bis-Tris Nu-PAGE gel (Invitrogen Corporation, CA) using MES buffer. The antibodies were used against AnxA2 (Mouse monoclonal, BD Biosciences, CA), Her-2, Akt, phosphorylated Akt (p-Akt; Ser473), STAT-3, pSTAT-3, apoptosis proteins (Cell Signaling Technologies, MA), extracellular signal-regulated kinase 1/2 (ERK1/2), phosphorylated ERK1/2 (BD Biosciences, CA), VEGF, uPA (R&D Technology, MN), Bcl-2, Bcl-xl, Bax-α, GAPDH (Santa Cruz Biotechnology, CA) and PGK [39]. Appropriate secondary antibodies conjugated to horseradish peroxidase (Promega, WI) were incubated with respective membranes for 2 hr at RT. The membranes were developed using ECL plus (Amersham Pharmacia Biotech, IL) and the image was captured using α-imager Fluoretech HD2. Immunoblot for PGK, GAPDH or β-actin were considered as internal control for loading.

### RNA Interference

Small interfering RNAs (siRNA) against AnxA2 (Catalog No. L-010741-00-0005), Her-2 (Catalog No. L-003126-00-0005), and scrambled nonspecific siRNA (control, Catalog No. D-001810-10-05) were purchased from Dharmacon (Lafayette, CO) (See Text S1 for sequence). To obtain effective silencing of protein expression, we utilized the SMARTpool siRNA reagent, which is a combination of four SMART selection-designed siRNAs in a single pool. Transfection was done following the manufacturer’s instruction using the Dharmafect 1 transfection reagent (Dharmacon).

### RNA Isolation and qPCR Assays

Total RNA was isolated using the Trizol solution (Invitrogen, Carlsbad, CA) according to the protocol provided by the manufacturer. Total RNA (1 µg) was reverse transcribed using oligo (dT) primers and SuperScript III RT (Invitrogen, Carlsbad, CA) in a total volume of 20 µl. qPCRs were performed with Platinum Taq DNA polymerase with integrated UDG carryover prevention technology and SYBR Green I fluorescent dye (Invitrogen, Carlsbad, CA), as described by the manufacturer in a final volume of 50 µl in an Eppendorf Realplex2 Master Cycler. PCR reaction set up used was 2 minutes 50°C for one cycle followed by 2 minutes at 95°C and 40 cycles of 30 s at 95°C, 30 s at 54°C, and 30 s at 68°C. The primer sequences used are as follows. AnxA2, Forward: 5′ TAA CTT TGA TGC TGA GCG GG 3′, Reverse: 5′ TAA TTT CCT GCA GCT CCT GG 3′; Her-2, Forward: 5′ CCT GTG CCC ACT ATA AGG AC 3′, Reverse: 5′ AGC TTC CGC ATC GTG TA 3′; and GAPDH Forward: 5′ GAG CGA GAT CCC TCC AAA 3′, Reverse: 5′ ACT GTG GTC ATG AGT CCT TC 3′.

### Cell Fractionation and Immunoprecipitation

MDA-MB-231 cells after respective treatment were lysed using hypotonic buffer, mixed and passed through a 20 gauge needle 15 to 20 times. Protein concentration was estimated using MicroBCA method following centrifugation to pellet cell debris at 5000 g. Equal amount of protein was taken from multiple treatments and subjected to ultracentrifugation at 72000rpm (100,000 g) for 30–60 minutes in an ultracentrifuge (Rotor – TLA 100.3) at 8°C. Following centrifugation, the supernatant (cytosolic fraction) was transferred into a fresh tube and the pellet washed with PBS and subjected to 30 minutes of ultracentrifugation. The pellet (membrane fraction) obtained was re-suspended in hypotonic buffer. These fractions were used to immunoprecipitate EGFR and AnxA2 using respective antibodies as previously described [38]. Quality of the membrane and cytosolic fractions were analyzed by blotting with Na+/K+ ATPase (mouse monoclonal antibody from Developmental Studies Hybridoma Bank, IA) and GAPDH (mouse monoclonal antibody, Santa Cruz Biotechnology, CA).

### Knockdown of AnxA2 in MDA-MB-231 and JIMT-1 Cells by Lentiviral Mediated pGIPZ shRNA

The shRNA specific for the AnxA2 gene was acquired from Open Biosystems, CO (Catalog No. RHS4430-101067266. See Text S1 for details). The AnxA2–specific shRNA was cloned in the pGIPZ lentiviral expression vector. The shRNA-containing lentiviral vector was transfected with lentiviral packaging into HEK-293T cells to produce shRNA carrying lentivirus particles. Culture supernatants were collected at 24 and 48 h after transfection and filtered through 0.45-µm membranes to generate cell-free virus supernatant. MDA-MB-231 and JIMT-1 cells were infected by the resulting viral particles, and positive clones were selected and maintained in puromycin (1–5 µg/mL). pGIPZ lentivirus of a non-silencing shRNA control with no homology to known mammalian genes was used as the negative control for the knockdown experiment. The resulting stable cell lines are called MDA-MB-231 AnxA2 sh and vector control MDA-MB-231 and JIMT-1 AnxA2sh and JIMT-1 vector control respectively.

### Focus Formation Assay

JIMT-1 AnxA2sh and JIMT-1 vector control cells were plated in DMEM/F12 containing 2% FCS at density of 1×10^4^ cells/60 mm plate. The medium was changed on alternate days. After 2 weeks of seeding, foci were stained with 0.5% crystal violet in 20% methanol and counted. Simultaneously, the crystal violet stain was washed three times in PBS and the adhered stained cells were solubilized with 1% SDS. The plates were agitated in the orbital shaker until the uniform colored solution is formed. The absorbance reading was taken at 570 nm. Multiple experimental readings were taken to correlate the test value with the control.

### Apoptosis Assay

Apoptosis in MDA-MB-231 cells after AnxA2 antibody treatment was analyzed by Vybrant apoptosis assay (Invitrogen Corporation, Carlsbad, CA) using flow cytometry (Cytomics FC 500 series flow cytometer, Beckman Coulter) measurements following manufacturer’s instructions.

### In vitro Tumor Invasion Assay

The invasive capacity of vector control and AnxA2sh JIMT-1 stable cells were tested using in vitro invasion assays (Becton Dickinson Bio-Coat Matrigel Invasion Chamber). The cells were seeded (2.5×10^4^) on each well of a 24-well, matrigel-coated invasion plate in serum-free DMEM-F12 basal medium. The cells were induced to invade toward a chemoattractant placed in the lower chamber (10% FBS containing DMEM-F12). After incubation for 24 hrs, the non-invading cells were removed from the upper surface of the invasion membrane and the cells on the lower surface were stained with BD Calcein AM Fluorescent dye (Molecular probes, Carlsbad, CA) incubated at 37°C for 1 hour and readings were taken with 494/517 filter in Synergy 2 Biotek plate reader. Growth medium incubated without any cells in the invasion system was used as blank reading and three independent experimental readings were used to calculate the mean fold change of invasion by determining the ratio of fluorescent reading-test group to fluorescence reading-vector control group.

### Other Methods

TIRF microscopy, cell viability assay and wound-healing assay were performed as described earlier [38], [40].

### Statistical Analysis

Statistical analysis was performed using SPSS Version of Windows. A paired t test and χ2 test were used to analyze the AnxA2 and Her-2 staining in various breast cancer samples. For other functional assays, GraphPad Prism 4.02 software (San Diego, CA) was used. The ImageJ software was used for immunohistochemistry analysis and densitometric analysis of western blots which was normalized with respective loading controls. One sample t-test was performed and p-values of <0.01 were considered to be significant.

## Results

### AnxA2 is Overexpressed in Her-2 Negative Breast Cancer

**Figure 1:**
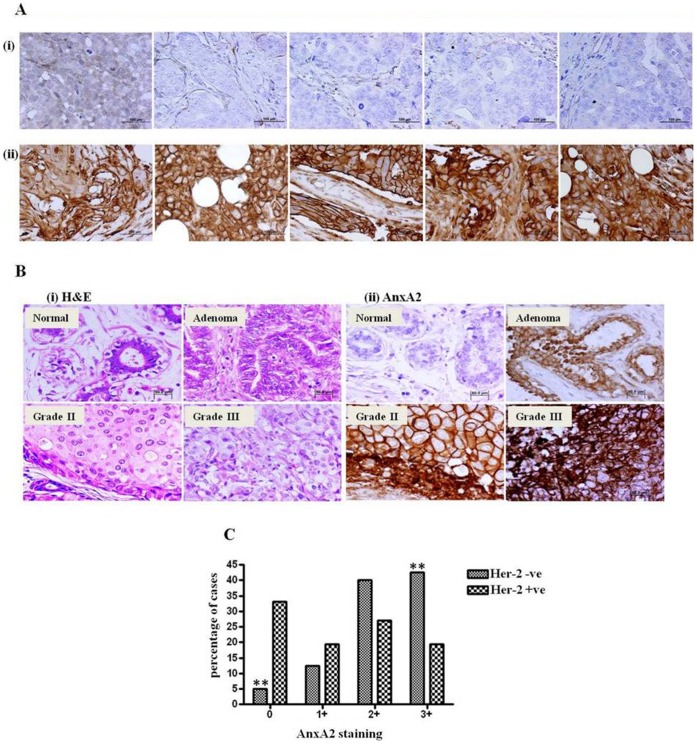
Immunohistochemical analysis of AnxA2 in Her-2 amplified and Her-2 negative/basal breast cancer tissue specimens. (**A**) Representative images from five different Her-2 amplified(i) and Her-2 negative(ii) breast cancer specimens showing expression status of AnxA2 and Her-2. Paraffin embedded tissue sections were stained with AnxA2 monoclonal antibody showing ‘3+’ expression (strong membranous staining in >30% cells) in Her-2 negative breast cancer tissue sections and ‘0’ expression (null staining) in Her-2 amplified breast cancer specimens. (Scale bar = 100 µm) (**B**) AnxA2 immunopositivity increases with the progression of the Her-2 negative breast cancer. Four histological subtypes of tissue sections (Normal, Adenoma, Grade II, Grade III) were stained with hematoxylin and eosin (H&E) (i) and AnxA2 monoclonal antibody (ii). Normal breast tissue section was null (0) for AnxA2 staining, adenoma showed increased membrane staining in cancer cells (1+, 0–10% cells) and grade II with intense diffused staining (2+, <30% cells) and high intensity of staining in the cells as well as stroma of poorly differentiated grade III (3+, >30–100% cells) Her-2 negative breast cancer. All images are taken in 100X magnification. (Scale bar = 50 µm) **(C)** Bar diagram showing the correlation of AnxA2 expression status between Her-2 negative and Her-2 amplified breast cancer cases. Very low percentage of Her-2 null cases demonstrated low AnxA2 expression leaving higher percentage (>80%) showing increased level of AnxA2 expression. Similarly inverse correlation was observed with Her-2 amplified breast cancer (Chi square P value = 0.005).

Tissue microarray and paraffin embedded tissue specimens were analyzed by immunohistochemistry (IHC) for Her-2 and AnxA2 expression. Representative IHC images of AnxA2 in Her-2 amplified and null patients are shown in Figure 1A. Her-2 amplified specimen (Figure 1Ai) showed weak staining of AnxA2 (0–1+, 0–10% cells showed very faint staining) while Her-2 null/basal breast cancer specimen demonstrated strong membrane expression of AnxA2 (Figure 1Aii, >3+, >30% cells with strong membranous staining). We found that the fraction of AnxA2 positive area in these sections were 3.8±2.49 and 61±11.07 for Her-2 null and amplified patients samples respectively (Figure S1A). Summarized data indicate that there is an inverse correlation of Her-2 amplification and AnxA2 expression (Table 1, p<0.0006). As represented in Table 1, ER and PR expression are not significantly associated with AnxA2 expression. Figure 1C represents the analysis of percentage of cases assayed with Her-2 status against AnxA2 staining intensities. Among the Her-2 negative breast cancer cases studied, >85% showed intense staining of AnxA2 (2+ or 3+), while 10% exhibited 1+ and <5% represented ‘0’ AnxA2 staining pattern. Conversely, AnxA2 expression was very low in a large proportion of Her-2 positive cases (Chi-square p-value = 0.005). We then tested if AnxA2 expression (staining intensity) increased with the progression of Her-2 negative breast cancer. As represented in Figure 1B, while some vessel walls in normal tissue specimens displayed light brown staining of AnxA2, the expression progressively increased in adenoma and early stages of breast cancer. Adenosis displayed mild membranous and cytosolic staining (1+) and this pattern became more intense (moderate staining, 2+) in grade II and significantly higher (3+ staining) in poorly differentiated grade III cancer (Table 2, p<0.007). Among the cases we analyzed, we saw that AnxA2 expression increased with progression of Her-2 negative breast cancer. We confirmed these findings by quantitative analysis of images using ImageJ software (Figure S1B).

**Table 1:** Association of AnxA2 staining with known biomarker(s) ER, PR and Her-2 expression status.

Biomarker Status	N	AnxA2(+)	AnxA2(−)	P value
**Her-2 (+)**	30	11	19	0.0006
**Her-2 (−)**	34	29	5	
**ER (+)**	33	20	13	0.9845
**ER (−)**	31	20	11	
**PR (+)**	30	17	13	0.5178
**PR (−)**	34	23	11	

**Table 2:** AnxA2 staining pattern with breast cancer progression.

Cancer Stage	Negative/Basal Staining of AnxA2	Positive/Intense Staining of AnxA2	Total
Normal	9	3	12
Adenoma	1	11	12
Grade I–II	12*	14	26
Grade III	13*	25	38
	34	54	88

P<0.007.

AnxA2 expression was significantly associated with the progression of the breast cancer except in Her-2 amplified cases*.

### AnxA2 Expression is Inversely Correlated with Her-2 Status in Breast Cancer Cell Lines

**Figure 2:**
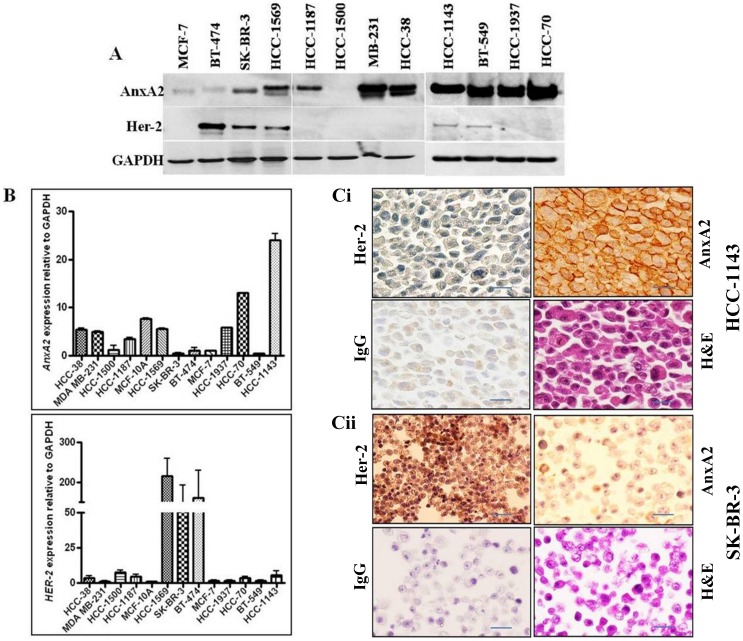
AnxA2 and Her-2 expression in breast cancer cell lines. (**A**) AnxA2 and Her-2 protein was analyzed by Western blotting in a breast cancer cell lines panel including Her-2 amplified SK-BR-3, BT-474 and HCC-1569; Her-2 null/negative HCC-38, HCC-1143, MDA-MB-231, BT-549, HCC-70, HCC-1937 and ER expressing HCC-1187, MCF-7 and HCC-1500. Glyceraldehyde-3-Phosphate Dehydrogenase (GAPDH) was used as loading control. (**B**) qPCR analysis showing mRNA expression of AnxA2 and Her-2 in breast cancer cell lines. Total RNA isolated from different breast cancer cell lines were reverse transcribed and then cDNA samples were PCR amplified. The amplification of AnxA2 was compared against the endogenous control GAPDH. The numbers shown are quantitative fold change. (**C**) Immunohistochemical analysis of AnxA2 and Her-2 in paraffin embedded HCC-1143 (i) and SK-BR-3 (ii) cell clots. Paraffin embedded blocks with cell clots were cut into serial 5 µM sections and were mounted onto superfrost TM adhesive-coated slides. The slides were processed for immunohistochemistry using AnxA2 (BD Biosciences, CA) and Her-2 (Cell Signaling, MA) antibodies. Mouse IgG was used as negative control. Immunostaining was performed with a kit from Vector laboratories using DAB solution. All images are 100X original magnification. All the three analyses, Western blot, qPCR analysis of mRNA and immunohistochemical analysis revealed that expression of AnxA2 is higher in breast cancer cells which express null/basal Her-2. (Scale bar = 50 µm).

We found an inverse correlation of Her-2 and AnxA2 expression in clinical specimens of breast cancer patients, which was consistent with the expression profile in breast cancer cell lines. The expression pattern of established biomarkers in breast cancer cell lines is shown in Table 3. Immunoblot analysis confirmed the immunohistochemistry and qPCR data showing mRNA expression pattern correlated with protein expression (Figure 2A and 2B). Immunohistochemical analysis of Her-2 negative cell line HCC-1143 demonstrated intense membranous staining of AnxA2 and weak staining of Her-2. In contrast, SK-BR-3 cells showed intense staining of Her-2 on the membrane compared to very weak staining of AnxA2 (Figure 2C), further supporting the reciprocal correlation between AnxA2 and Her-2 expression in breast cancer.

**Table 3:** Hormone receptors PR, ER and Her-2 status of the breast cancer cell lines used.

Cell lines	PR Status	ER Status	HER2 Status
MCF-10A	–	–	–
HCC-38	–	–	–
HCC-1143	–	–	B/−
MDA-MB-231	–	–	–
BT-549	–	–	B/−
HCC-70	–	–	–
HCC-1937	–	–	–
MCF-7	B	B	B
HCC-1187	–	B	–
HCC-1500	B	B	B
BT-474	+	+	+++
SK-BR-3	–	–	+++
HCC-1569	–	–	+++

B basal. –null. +Gene amplification.

### Her-2 Downregulation Induces AnxA2 Expression in Her-2 Amplified Cells

**Figure 3:**
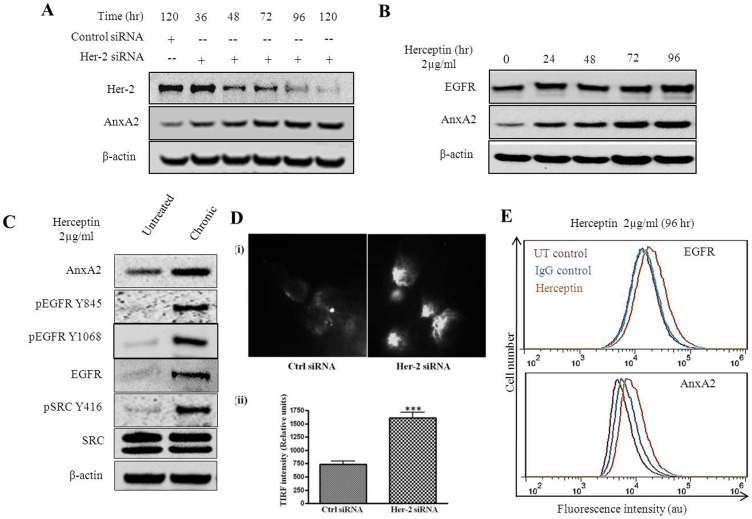
Effect of Her-2 knockdown or Herceptin treatment in Her-2 amplified cell line. (**A**) Her-2 amplified SK-BR-3 cells were transfected with nonspecific siRNA or Her-2 siRNA. Cells were collected at different time points after transfection, lysed and analyzed by Western blotting with Her-2 and AnxA2 antibodies. (**B**) SK-BR-3 cells were treated with Herceptin (2 µg/ml) at different time intervals as indicated. After the respective treatments, cells were lysed and the expression of EGFR, AnxA2 and PGK by Western blotting. Blots shown are from one representative experiment and each experiment was repeated three times to ensure reproducibility. (**C**) SK-BR-3 cells were treated for eight weeks with Herceptin (2 µg/ml), by changing the media and the treatment twice a week and were subsequently processed for Western blotting alond with the untreated control cells. (**D**) SK-BR-3 cells were grown on coverslips, transfected with nonspecific siRNA or Her-2 siRNA. After 72 hrs of transfection, cells were fixed without permeabilization and treated with anti-AnxA2 antibody. Coverslips were mounted on special coverslips and were visualized by TIRF Microscopy. White spot reflection as seen in the images represent the increased membrane localization of AnxA2 in Her-2 siRNA transfected cells compared to control (i) The figure shows a representative image from multiple experiments. All TIRF images are taken in 60X oil immersion magnification. TIRF intensity of cell surface AnxA2 in 12 different microscopic fields was quantified. Mean values of (±SD) of the Ctrl siRNA and Her-2 siRNA treatment is shown in the bar graph with statistical significance of ***p<0.0001 for student’s t-test (ii). (**E**) SK-BR-3 cells were treated with either Herceptin or isotype control for 96 hrs and trypsinized, washed and fixed with 4% paraformaldehyde. These cells were incubated with 3% BSA in PBS for blocking followed by incubation with primary antibody against EGFR and AnxA2 (Santa Cruz Biotechnology, CA). The cells were then washed with PBS twice and incubated with Alexa 488 labeled secondary antibodies for 2 hrs at room temperature, washed and subjected to flow cytometry.

We investigated whether the expression of AnxA2 can be induced by down regulation of Her-2. We transfected Her-2 amplified SK-BR-3 cells with Her-2 siRNA (90.14% knockdown in 120 hr) and found an induction of AnxA2 protein levels (2.42 fold in 120 hr) in a time dependent manner (Figure 3A). It is established that EGFR and Src expression is upregulated in Her-2 negative and Herceptin-resistant breast cancers [9], [13], [14], [41], [42]. To validate whether AnxA2 is also co-regulated with these proteins, we treated SK-BR-3 cells with Herceptin (2 µg/ml) and found a time dependent increase in EGFR (1.3 fold in 96 hr) and AnxA2 expression (3.3 fold in 96 hr)(Figure 3B). We could also replicate these results in a Her-2 amplified cell line such as HCC-1569 using an antibody against Her-2 receptor (Figure S2). As suggested in previous reports, we expected an increase in phosphorylated Src (Y416) (6 fold) upon Herceptin treatment. However, this was only upon chronic treatment of Herceptin (8 weeks) in Her-2 amplified SK-BR-3 cells. We also found significant increase in protein levels of EGFR (7 fold), pEGFR (Y1068 and Y845) as well as AnxA2 (1.9 fold) upon chronic treatment (Figure 3C). This could be possible as Src is associated with Her-2 receptor and its phosphorylation is inhibited with Herceptin treatment. However, when the EGFR is continuously activated (Figure 3C), it might phosphorylate Src at Y416 position to activate the downstream signaling pathway. It is well established that AnxA2 is a binding partner of v-Src and facilitate recruitment of v-Src to the focal adhesions [33]. Recruitment of AnxA2 to inner leaflet of plasma membrane leads to its increased cell surface expression via the exosomal pathway [25]. This surface-localized AnxA2, along with its binding partner, then leads to degradation of extracellular matrix and helps in migration and invasion of cancer cells [25], [43], [44]. Therefore, the presence of AnxA2 at the membrane is important for its various functions. We examined the cell surface localization of AnxA2 upon siRNA-mediated downregulation of Her-2 using total internal reflection fluorescence microscopy (TIRFM) [45]. In TIRFM, membrane surface proteins can be detected by the decaying light intensity as it penetrates the cell/substratum. Staining of unpermeabilized SK-BR-3 cells with AnxA2 antibody tagged to Alexa fluorophore-568 showed numerous dense spots of AnxA2 protein on the membrane surface (Figure 3Di) after Her-2 siRNA treatment compared to control siRNA treatment. We quantified the intensity of TIRF signal in 12 different microscopic fields demonstrating a significant increase in surface translocation of AnxA2 upon Her-2 downregulation (Figure 3Dii). We validated these results as well as upregulation of EGFR receptor by flow cytometry (Figure 3E). These results indicate a predominant membrane localization of AnxA2 upon downregulation of Her-2 or its downstream signaling.

### AnxA2 is Modulates EGFR Mediated cell Survival Pathways and Cancer Cell Migration

**Figure 4:**
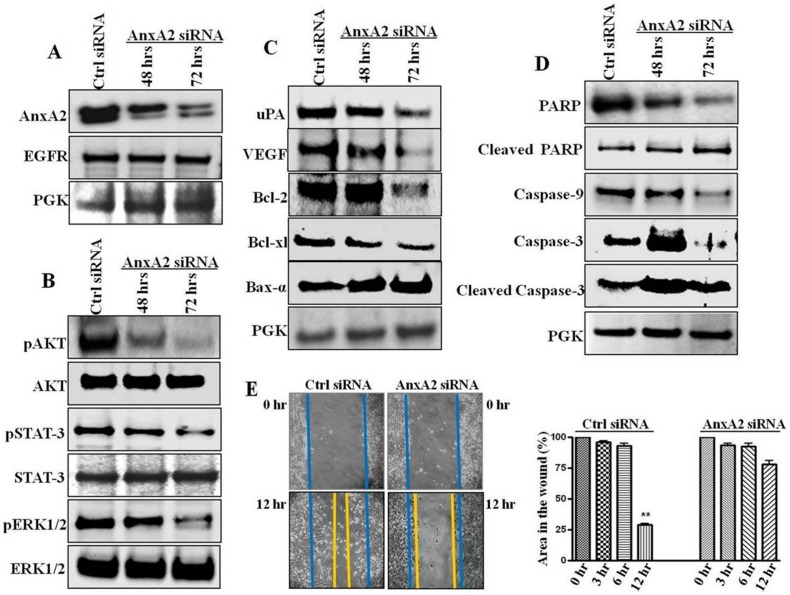
Effect of AnxA2 knockdown in Her-2 null breast cancer cells. MDA-MB-231 cells were transfected with Dharmacon smart pool AnxA2 siRNA and nonspecific scrambled siRNA. After 48 hrs and 72 hrs of transfection protein was harvested. (**A**) AnxA2 knock down and EGFR status was ascertained by Western blot. (**B**) Effect of AnxA2 downregulation on EGFR downstream signaling was analyzed by Western blotting with pERK1/2/ERK1/2, pSTAT-3/STAT-3 and p-AKT/AKT antibodies. (**C**) Metastatic proteins uPA and VEGF, anti-apoptotic proteins Bcl-2, Bcl-xl and pro-apoptotic Bax-α were analyzed after downregulating AnxA2 in MDA-MB-231 cells. (**D**) Activation of apoptotic caspases and PARP cleavage in AnxA2 siRNA treated MDA-MB-231 cells was analyzed by Western blotting of PARP, cleaved PARP, Caspases 3, 9 and respective cleaved caspases. (**E**) MDA-MB-231 cells were treated with AnxA2 siRNA/nonspecific siRNA for 48 hrs. Scratch was made using a pipet tip, after the wound formation plates were incubated and photographs were taken for different time intervals (0, 3, 6, 12 hrs). Representative pictures of 0 and 12 hrs are shown in the figure. Graph shows the Image J value of percentage wound closure ar different time intervals after wound formation. Error bars indicated the mean of three independent experiments; **p<0.001, relative to non specific siRNA treated cells.

The increase in EGFR, Src and AnxA2 levels upon Her-2 downregulation and inhibition led us to investigate the role of AnxA2 in EGFR signaling in TNBC subset of breast cancer, where EGFR plays a major role in survival, growth, migration and invasion of cancer cells [15], [16], [46], [47]. Phosphorylated AnxA2 has been shown to translocate to the cell membrane and associate with other membrane protein complexes where it plays a critical role in actin cytoskeletal rearrangement, cell migration and plasminolysis at the cell surface [26], [28], [33], [48]. After siRNA-mediated downregulation of AnxA2 (54% at 72 hrs) we did not see any change in total EGFR protein expression (from 1.00 to 1.01 fold) (Figure 4A). However, there was a significant reduction in levels of pErk1/2 (62%), pSTAT-3 (49%) and pAKT (43%) after 72 hrs (Figure 4B), without affecting the total protein levels of these molecules. Although there are other pathways included in activation of these proteins; later we confirmed the involvement of EGFR mediated signaling in these cells.

AnxA2 plays a role in angiogenesis [28] and extracellular matrix degradation [43], [44]. We investigated whether AnxA2 downregulation, along with decreased signaling pathway(s), affects invasive properties of TNBCs. Invasive basal breast cancer progression is facilitated by increased expression of serine protease uPA and VEGF [49], [50]. As shown in Figure 4C, AnxA2 siRNA treatment significantly decreased the expression of these proteins (decrease in uPA 61%; VEGF 69% within 72 hrs). To confirm this at the functional level, we performed a wound-healing assay. Indeed, AnxA2 expressing cells showed higher cellular motility and we found 75% wound closure as compared to control siRNA upon siRNA-mediated downregulation of AnxA2 (p<0.001). Figure 4E shows the representative images of wound closure after 12 hr of wound formation, which was made after 48 hrs of transfection (Figure 4E).

We speculated that if AnxA2 downregulates constitutive activation of STAT-3, it should attenuate the expression of Bcl-2 and Bcl-xL and augment Bax-α. As expected, AnxA2 downregulation significantly decreased the expression of anti-apoptotic Bcl-2 (44%) and Bcl-xL (69%) and induced the pro-apoptotic Bax-α (20%) (Figure 4C). To further evaluate the cytotoxic effect of AnxA2 downregulation, we analyzed PARP cleavage and the expression pattern of different caspases. In MDA-MB-231 cells, AnxA2 siRNA treatment activated caspase-mediated apoptosis resulting in significant increase in cleaved PARP (1.99 fold), cleaved caspase-3 (1.87 fold) and a decrease in caspase-9 (0.72 fold) (Figure 4D). These results suggest that AnxA2 plays a critical role in maintain activation of several signaling intermediates responsible fore cell survival, growth and migration.

### AnxA2 Downregulation Decreases Activation of Several Signaling Pathways in Herceptin-resistant JIMT-1 Cells

**Figure 5:**
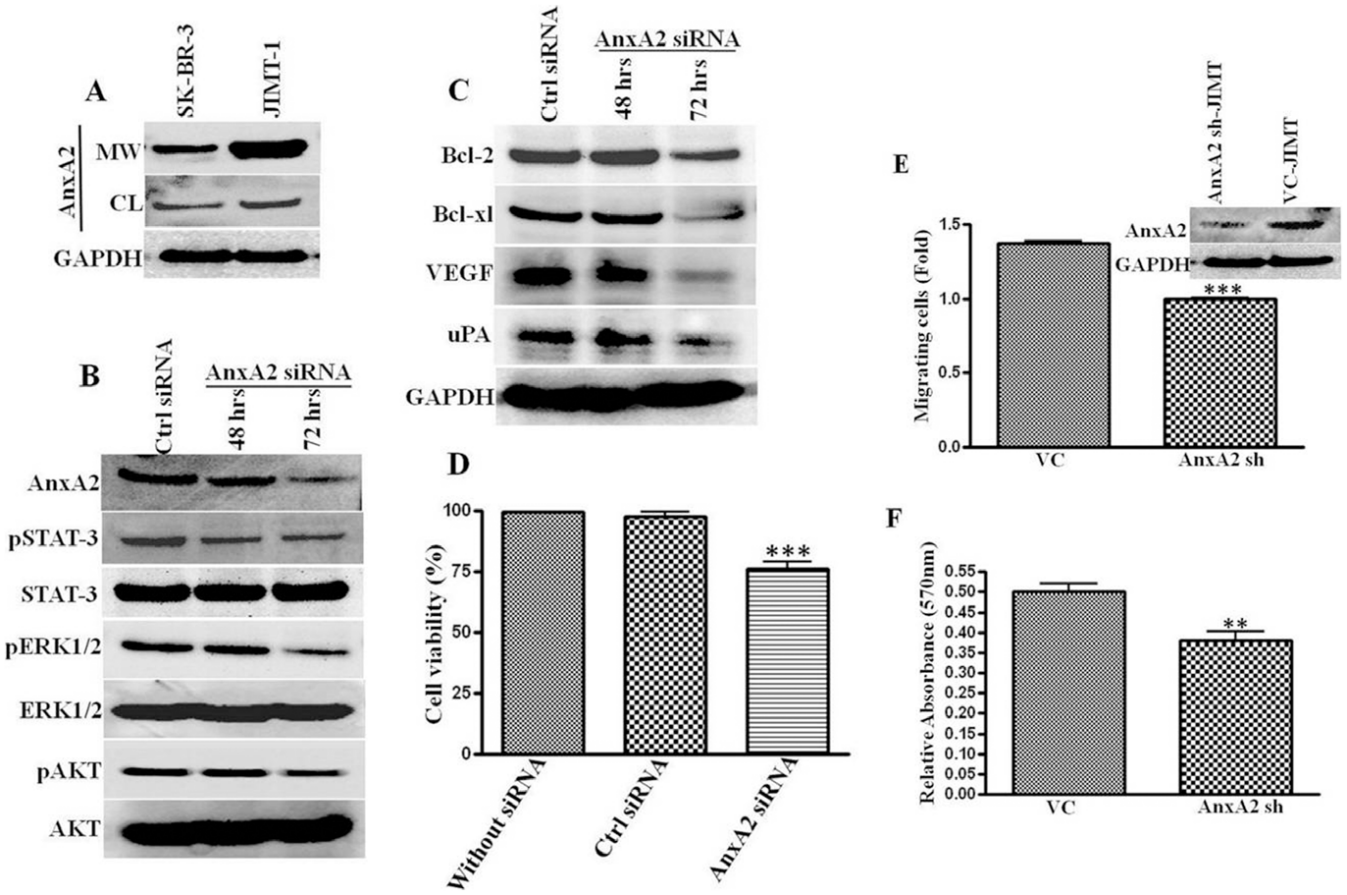
Effect of AnxA2 knockdown in Herceptin-resistant JIMT-1 cell line. (**A**) Confluent SK-BR-3 and JIMT-1 cells were washed with PBS and, EDTA. These washed and the cell lysate were analyzed for AnxA2 and GAPDH. JIMT-1 cell line demonstrated high expression of cytosolic and membrane surface AnxA2 compared to SK-BR-3. (**B**) AnxA2 expression in JIMT-1 cells was downregulated by AnxA2 siRNA treatment. The effect of AnxA2 knockdown on EGFR downstream signaling (pAKT/AKT, pSTAT-3/STAT-3 and pERK1/2/ERK1/2) was analyzed. (**C**) The antiapoptotic proteins Bcl-2, Bcl-xl and metastatic proteins uPA and VEGF were also analyzed by Western assay after AnxA2 downregulation in JIMT-1 cells. (**D**) The JIMT-1 cell viability was verified by MTT assay after 96 hrs of AnxA2 siRNA transfection and compared with control siRNA treatment (***p<0.0001). (**E**) pGIPZ vector control and AnxA2 shRNA expressing stable JIMT-1 cells were produced by lentiviral vector delivery and subsequent puromycin antibiotic selection as mentioned in methods. AnxA2 downregulation in these cell lines were confirmed and used to study the effect of AnxA2 knockdown on cell migration using tumor biocoat assay (BD Biosciences, CA). Approximately 2.5×10^4^ cells were seeded onto matrigel-coated tumor invasion chambers and allowed to migrate towards serum for 24 hrs at 37°C. The migrated cells were stained with Calcein AM and fluorescent reading was measured and fold change was quantified against vector control cells. The bar graph represents the mean of three independent experiments (±SD) *** p<0.0001 relative to vector control. *F,* Suppression of AnxA2 in JIMT-1 cells reduced foci formation. Vector control as well as AnxA2sh JIMT-1 cells were seeded and grown for 21 days (n = 3). Difference in the foci formation was quantified using crystal violet absorbance assay (**P<0.001, student’s t-test).

Resistance to Herceptin may arise due to de novo resistance or acquired resistance [9]. The de novo resistance is generally caused by genetic alteration in the Her-2 receptor, whereas acquired resistance is usually due to prolonged treatment leading to activation of rescue mechanisms or alternative survival pathways. Our data on increase in AnxA2 expression upon treatment with the Her-2 antibody as well as prolonged Herceptin therapy (Figure 3C), and other reports suggesting involvement of AnxA2 in cancer recurrence during the neoadjuvant therapy, chemotherapy and radiotherapy [51]–[55] suggested the role of AnxA2 in Herceptin resistance. To study this, we used JIMT-1 cells (isolated from a Herceptin-resistant breast cancer patient) and screened for AnxA2 expression. We found relatively high cytosolic AnxA2 with abundant membrane localization (2.21 fold) of AnxA2 in JIMT-1 cell line compared to its Her-2 amplified counterpart (Figure 5A). Similar to MDA-MB-231 cells, AnxA2 downregulation in JIMT-1 cells showed a decreased activation of signaling molecules (pSTAT−3 42%; pERK1/2 55%; pAKT 48%), antiapoptotic (Bcl−2 14%; Bcl-xl 43%) and metastatic markers (VEGF 58%; uPA 22%) (Figure 5B and 5C). Moreover, AnxA2 siRNA treatment significantly decreased the cell viability of JIMT-1 cells as compared to control siRNA treatment (p<0.0001, student’s t test). To determine the role of AnxA2 in cell migration of JIMT-1 cells, we used lentiviral delivery of sh-AnxA2 and vector control followed by tumor biocoat migration assay. We found a significant decrease in migration of JIMT-1 cells upon shRNA mediated AnxA2 downregulation as compared respective control cells (Figure 5E, p<0.0001; student’s t test). In addition, we evaluated the effect of AnxA2 knockdown on cancer cell growth and colony formation by a focus formation assay and found significantly fewer foci as compared to vector control (Figure 5F, p<0.005). These results demonstrate that AnxA2 plays a critical role in activation and/or modulation of different survival pathways, growth and migration of Herceptin-resistant JIMT-1 cells. These results also suggest that AnxA2 could be used as a predictive biomarker and a therapeutic target in Herceptin-resistant cells.

### AnxA2 Associates with Membrane EGFR Complex and is Essential for Ligand Induced EGFR Mediated Downstream Signaling

**Figure 6:**
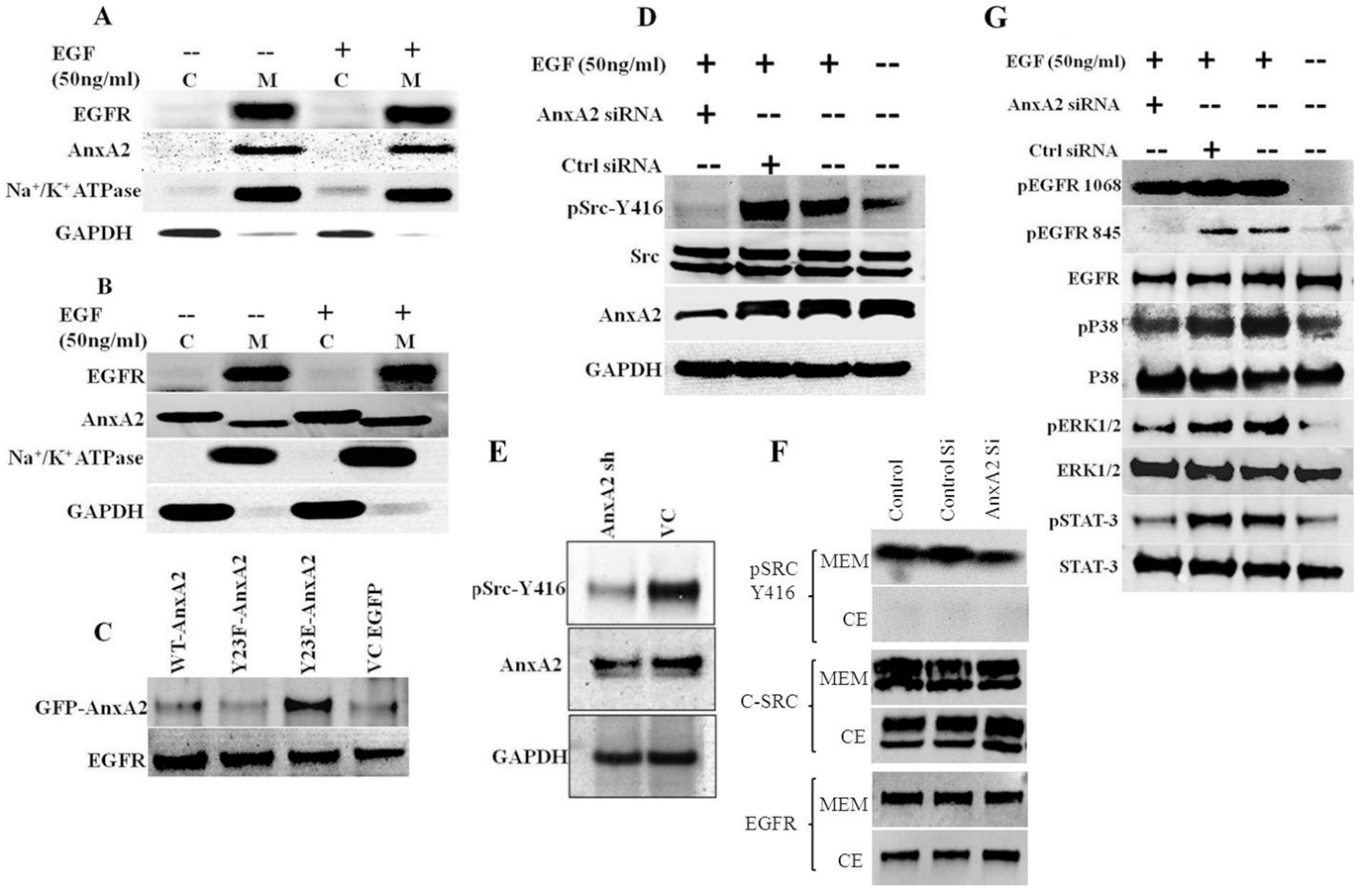
Role of AnxA2 in EGFR signaling complex in Her-2 negative breast cancer. (**A & B**) Immunoprecipitation assay to demonstrate AnxA2-EGFR association at the cell membrane. MDA-MB-231 cells at 80% confluence were serum starved for 12 hrs, treated with or without EGF (50 ng/ml) for 20 minutes. Cells were lysed in hypotonic lysis buffer, membrane and cytosolic fractions were separated by ultracentrifugation. These fractions were used for immunoprecipitation with EGFR (A) and AnxA2 antibody (B). The immunoprecipitated product was blotted for EGFR, AnxA2 and the purification of the membrane and cytosolic fractions were analyzed by blotting with Na+/K+ ATPase and GAPDH. (**C**) Phosphomimic GFP-AnxA2 Y23E, phosphomutant GFP-AnxA2 Y23F and wild type AnxA2 in pEGFP-N1 vector and empty vector control plasmids were transiently transfected to MDA-MB-231 cells. After 60 hrs of transfection, cells were serum starved for 12 hrs. These serum starved cells were lysed and immunoprecipitated with EGFR antibody and blotted with GFP antibody for the fusion GFP-AnxA2 expression. In the serum deprived cells, only phosphomimic Y23E GFP-AnxA2 could associate with EGFR. (**D**) MDA-MB-231cells were transfected with control siRNA and AnxA2 siRNA for 60 hrs. After overnight serum starvation, cells were treated with/without EGF (50 ng/ml) for 20 minutes. Cells were lysed and Western blot analysis was performed with AnxA2 antibody to confirm the downregulation. The same lysate was assayed for pSrcY-416 and Src. (**E**) The stable AnxA2 sh and pGIPZ vector control cell lines were produced in MDA-MB-231 parental cells using Lenti-viral delivery of pGIPZ vector containing AnxA2 shRNA and puromycin antibiotic selection. These stable cell lines were analyzed for AnxA2, pSrcY-416 and GAPDH. (**F**) MDA-MB-231 cells were transfected with AnxA2 siRNA and the cells were harvested at 72 hrs by trypsin from serum containing medium. The cells were lysed and processed for cytosolic and membrane protein fraction using Pierce Mem-PER membrane protein extraction kit. *G.* AnxA2 siRNA and EGF treated MDA-MB-231 cell lysates were analyzed for pEGFR Y-1068, pEGFR Y-845 and normalized with EGFR. Similarly, downstream signaling molecules like pERK1/2/ERK1/2, pP38/P38, pSTAT-3/STAT3 were analyzed using respective antibodies.

Our findings suggest the co-activation of EGFR with AnxA2 in Hereceptin-resistant cells lines. Hence, we investigated whether AnxA2 is required for EGFR-mediated downstream pathways. AnxA2 is involved in translocation of Src to the plasma membrane [33]and growth factors like EGF and insulin are known to induce PI 3 kinase and Src-mediated phosphorylation and activation of AnxA2 [26], [56], [57]. Subsequently, AnxA2 is known to be involved in actin polymerization, which consequently also helps in migration of cancer cells [26]. Our recent work has established that the phosphorylated AnxA2 at Y23 residue is preferentially localized to the lipid raft domains of the plasma membrane [25]. The lipid raft associated AnxA2, along with other annexin family proteins such as Annexin A6, leads to the formation of endocytic vesicles and help in protein sorting [34], [35]. Considering the multitude of functions of AnxA2 in signaling complexes, we suspected that AnxA2 may be present in the EGFR membrane protein complex. We conducted immunoprecipitation experiments with membrane and cytosolic extracts of MDA-MB-231 cells with or without EGF activation. Figure 6A shows that EGFR antibody could pull down AnxA2 in the membrane fraction. Similarly EGFR is immunoprecipitated with AnxA2 antibody (Figure 6B). Interestingly, this interaction was independent of EGF stimulation, which suggests that AnxA2 is inherently present in the EGFR receptor protein complex and not transiently recruited upon stimulation.

To further assess whether the phosphorylation of AnxA2, which is recruited to the plasma membrane and lipid rafts, is necessary for interacting with EGFR, we used different AnxA2 mutant constructs. We transfected GFP-AnxA2 Y23F mutant, GFP-AnxA2Y23E phosphomimic, wild type GFP-AnxA2 and empty vector EGFP-N1 in MDA-MB-231 cells. The serum deprived cells, after 72 hrs of transfection, were analyzed for association of AnxA2 with EGFR by immunoprecipitation. As shown in Figure 6C, only phosphomimic Y23E-AnxA2 translocated to membrane and associated with EGFR. The wild type AnxA2 was minimally phosphorylated in serum deprived condition and hence showed very minimal interaction.

As mentioned before, Src is one of major regulators of EGFR downstream signaling [9], [16], [17]. AnxA2 is a known interacting partner of Src [33]. Studies have also demonstrated the cross-regulation of EGFR and Src. While, EGFR can phosphorylate Src at Y416 position, oncogenic Src (v-Src) can lead to phosphorylation of EGFR at Y845 site [58]. It is thus conceivable that these two proteins are present in a complex with each other. Previous reports about the involvement of AnxA2 in transportation of Src to the membrane led us to investigate whether AnxA2 downregulation interferes with EGF-induced phosphorylation of Src by EGFR. As shown in figure 6D, AnxA2 knockdown (59%) drastically reduces phosphorylation (64%) of Src at Y416, without altering the total Src expression. This was confirmed using AnxA2 shRNA expressing stable MDA-MB-231 cells (Figure 6E). To further validate this, we separated the membrane and cytosolic fractions of MDA-MB-231 cells transfected with AnxA2 siRNA (with serum). As expected, we found lower levels of pSrc in membrane fraction without any change in total Src (Figure 6F). Previous reports have shown that AnxA2 is involved in translocation of Src within the plasma membrane domains [33]. However, there could be other mechanisms involved in this effect. Further, we wanted to validate whether the ligand-induced EGFR downstream signaling would be affected by siRNA-mediated downregulation of AnxA2. We found decreased activation of signaling molecules like pERK1/2 (71%), pP38 (16%), pSTAT-3 (89%) upon AnxA2 downregulation as compared to only EGF treated or control siRNA treatment (Figure 6G). These results suggest that AnxA2 is important in regulation of EGFR downstream signaling. From these observations, we conclude that AnxA2 is involved in EGFR downstream signaling, either directly or indirectly, in a Src dependent manner and significantly contributes to the survival, growth and progression of Her-2 negative breast cancer.

## Discussion

This study reports a significant finding about the potential of AnxA2 as a diagnostic and/or prognostic marker as well as a therapeutic target in Her-2 negative, Herceptin-resistant and TNBC subset of breast cancer. The immunohistochemical analysis of breast cancer clinical samples and of different clinical grades confirms a negative correlation of AnxA2 with Her-2 expression (Figure 1). The hormone receptor status such as Her-2, ER and PR has been conventionally used in categorizing the breast cancer subset and the consequent therapeutic options for these patients. However, recent evidence has clearly established the heterogeneity of cancer and consequently argues for use of novel biomarker analysis [9]. The expression of AnxA2 in Her-2 negative and Herceptin-resistant subsets, as well as other molecular markers such as EGFR, could be used to define whether Herceptin can be used as a first line therapy for these patients. Moreover, our findings indicate that the expression of AnxA2 correlates with the aggressiveness of breast cancer and substantiates its prospects as a prognostic marker (Figures 1 and 2). We found that the surface expression of AnxA2 increases with acute or chronic treatment of Herceptin in a cell culture model. This also holds true in the Herceptin-resistant JIMT-1 cells (Figure 5). This suggests that AnxA2 could be used as a diagnostic and/or prognostic marker for acquired resistance against Herceptin. Recent evidence also suggests the use of a Src inhibitor along with Herceptin treatment increase therapeutic outcome in animal models in Herceptin-resistant breast cancer cells [9]. However, categorizing the Herceptin-resistant patients in a clinical setting to determine the best therapeutic regimen is still an unmet need. Further clinical should validate the potential use of AnxA2 as a diagnostic and/or prognostic tool in Her-2 negative, Herceptin-resistant and TNBC subsets of breast cancer.

Upregulation/activation of alternative survival proteins/pathways as a rescue mechanism by cancer cells upon molecularly targeted therapies has been proposed by several investigators. Here we report that AnxA2 is one of the proteins regulated with such survival proteins/pathways upon Herceptin therapy. We and other have shown that the expression of EGFR is upregulated in Herceptin-resistance and in TNBC subset of breast cancer (Figure 3B, 3C, and 3D). Moreover, we also confirmed overactivation of Src upon chronic treatment with Herceptin, which is consistent with other reports [9]. Although we are yet unaware of the mechanism by which the expression of AnxA2 is regulated along with these proteins, we are actively looking for both transcriptional and post-translational regulation of AnxA2 upon Herceptin treatment. Never-the-less, our study was extended to Herceptin-resistant JIMT-1 cells and MDA-MB-231 TNBC cells to delineate the role of AnxA2 in EGFR signaling and the underlaying mechanism (Figures 4 and 5). Our findings suggest that siRNA-mediated downregulation of AnxA2 leads to decreased activation of different survival proteins such as pAKT, pERK and pSTAT-3 (Figures 4 and 5). We also found that downregulation of AnxA2 in MDA-MB-231 and JIMT-1 cells leads to decreased cell proliferation. This could be attributed to the regulation of apoptotic proteins upon AnxA2 siRNA treatment. Moreover, we also showed that downregulation of AnxA2 leads to decreased migration of these cells and also decreased expression of migration associated genes (Figures 4 and 5). It is previously established that the surface AnxA2 acts as a catalytic center for tissue plasminogen activator (tPA) and helps in conversion of plasminogen to plasmin [27], [28]. These studies demonstrate the involvement of AnxA2 in inhibiting key signaling pathways, this leads to decreased migration and increased apoptosis, and its potential use as therapeutic target in Herceptin-resistant and TNBC subset.

Although, the effect of AnxA2 downregulation on several key signaling proteins was promising; it was equally important to delineate the mechanism by which AnxA2 regulates with these proteins. Previous reports have shown that EGFR and Src protein expression is increased in Herceptin resistance and TNBC cells [9]. Previous literature suggests the interaction of AnxA2 with EGFR and Src [33]–[35]. Our recent studies have shown that phosphorylation of AnxA2 at Y23 leads to its preferential localization in lipid raft domain of the cells [25]. Although controversial, it is known that EGFR is also located in lipid raft domains, which brings together several signaling proteins and activates the downstream signaling upon ligand stimulation. Indeed, we observed activation of Src kinase and EGFR phosphorylation at different sites upon chronic treatment with Herceptin (Figure 3C) and an increased membrane translocation in JIMT-1 cells (Figure 3D and Figure 5A). We showed that AnxA2, especially the phosphorylated one, interacts with EGFR in an EGF independent manner in MDA-MB-231 cells. These results suggest that Herceptin therapy causes activation of the EGFR-Src mediated alternative survival pathway and via AnxA2 (Figures 4, 5 and 6). AnxA2 is a multifunctional protein and is phosphorylated by several kinases such as protein kinase C (PKC), phospholipase C (PLC), as well as Src kinase. There are several reports suggesting the interaction and a crucial functional interplay of AnxA2 and Src protein at the membrane. AnxA2 is known to help v-Src mediated actin-cytoskeletal rearrangement and also acts a key regulator of v-Src in its localization in plasma membrane microdomains and endosomal membrane components. Results have indicated that AnxA2 regulates the activation of Src and modulate its downstream signaling [33]. On the other hand, it is also established that Src acts as one of the key mediators of EGFR signaling [17], . Our data suggest that although the translocation of Src is not affected by AnxA2 downregulation (Figure 6F), there is decreased activation of Src at the membrane, which could be due to lack of its accessibility to EGFR receptor complexes. However, the extent of the decrease was lower in these cells as the cells were grown in serum containing culture medium and were not starved before collection.

We also found decreased activation of several other signaling molecules in EGF stimulated cell lysates where AnxA2 is downregulated. Surprisingly, the ligand-dependent Y845 phosphorylation of EGFR was also markedly reduced upon AnxA2 downregulation. It is previously known that EGFR can phosphorylate Src at Y416 site, whereas activated Src can phosphorylate EGFR at Y845 site in a defined cellular context [58]. However, the activity of Src depends on its localization to the plasma membrane microdomains. It has been shown that depletion of cholesterol by methyl-β-cyclodextrin leads to decreased plasma membrane localization of Src [33] and it also impairs localization of AnxA2 to the lipid raft domains [25]. These studies along with our findings suggest that the spatiotemporal components define the localization and signaling downstream to EGFR and Src which are hampered by AnxA2 downregulation. Our data suggest a critical role of AnxA2 in EGFR signaling. However, further studies are needed to define the actual sequence of events, which is beyond scope of the present work.

Collectively, the increase in active Src (pSrc Y416), EGFR and AnxA2 upon Herceptin therapy and decrease in phosphorylation of Src and EGFR suggests that the cancer cells reprogram themselves by upregulation of a group of proteins which work together to overcome any stress such as Herceptin therapy. This facilitates the development of resistance, which then leads to clinical relapse and progression of the disease. Therefore, AnxA2 could potentially be used as a diagnostic marker as well as therapeutic target in Her-2 negative cancers.

## Supporting Information

Figure S1 **Inverse and positive correlation of AnxA2 expression in Her-2 negative cancer and their progression respectively. (A)** Fraction of AnxA2 positive area in Her-2 amplified and Her-2 negative breast cancer. **(B)** Fraction of AnxA2 positive area in different stage of Her-2 negative breast cancer cases. **(C)** Fraction of AnxA2 positive area in breast cancer cells. Quantitative analysis of Figure 2Ci and 2Cii.
(TIF)

Figure S2 **Her-2 downregulation in Her-2 amplified HCC-1569 cell line. (A)** Her-2 amplified HCC-1569 cells were transfected with nonspecific siRNA or Her-2 siRNA. After 72 hours from transfection, cells were lysed and analyzed by Western blotting with Her-2 and AnxA2 antibodies. **(B)** The HCC-1569 cells were treated with Her-2 antibody (2 µg/ml) for different time intervals. After the respective treatment, cells were lysed and analyzed the expression of EGFR, AnxA2 and PGK by Western blotting. Blots shown are from one representative experiment and each experiment was repeated three times to ensure reproducibility normalized with EGFR. Similarly downstream signaling molecules like pERK1/2/ERK1/2, pP38/P38, pSTAT-3/STAT3 were analyzed using respective antibodies.
(TIF)

Text S1 **Sequence of control, Her-2 and AnxA2 siRNA and AnxA2 shRNA.**
(DOC)
