# Correction: Reciprocal Regulation of Annexin A2 and EGFR with Her-2 in Her-2 Negative and Herceptin-Resistant Breast Cancer

**DOI:** 10.1371/annotation/ba62ae01-bf71-46c1-a45c-32692d3bb4ab

**Published:** 2012-09-07

**Authors:** Praveenkumar K. Shetty, Sanjay I. Thamake, Swati Biswas, Sonny L. Johansson, Jamboor K. Vishwanatha

There is error in author affiliations. The affiliation for PKS and SB are the current affiliations. All the work presented in this article was performed at University of North Texas Health Science Center at Fort Worth. Previous affiliation for PKS - Department of Molecular Biology and Immunology and Institute for Cancer Research, University of North Texas Health Science Center, Fort Worth, Texas; and for SB- Department of Biostatistics, School of Public Health, University of North Texas Health Science Center, Fort Worth, Texas. JKV is not affiliated with Department of Biochemistry, SDM College of Medical Sciences & Hospital, Dharwad, India.

