# Supplementary figures and images for: Reciprocal Regulation of Annexin A2 and EGFR with Her-2 in Her-2 Negative and Herceptin-Resistant Breast Cancer
Source: PLoS One. 2012 Sep 5;7(9):e44299. doi: 10.1371/journal.pone.0044299 (PMC3434131; doi:10.1371/journal.pone.0044299)

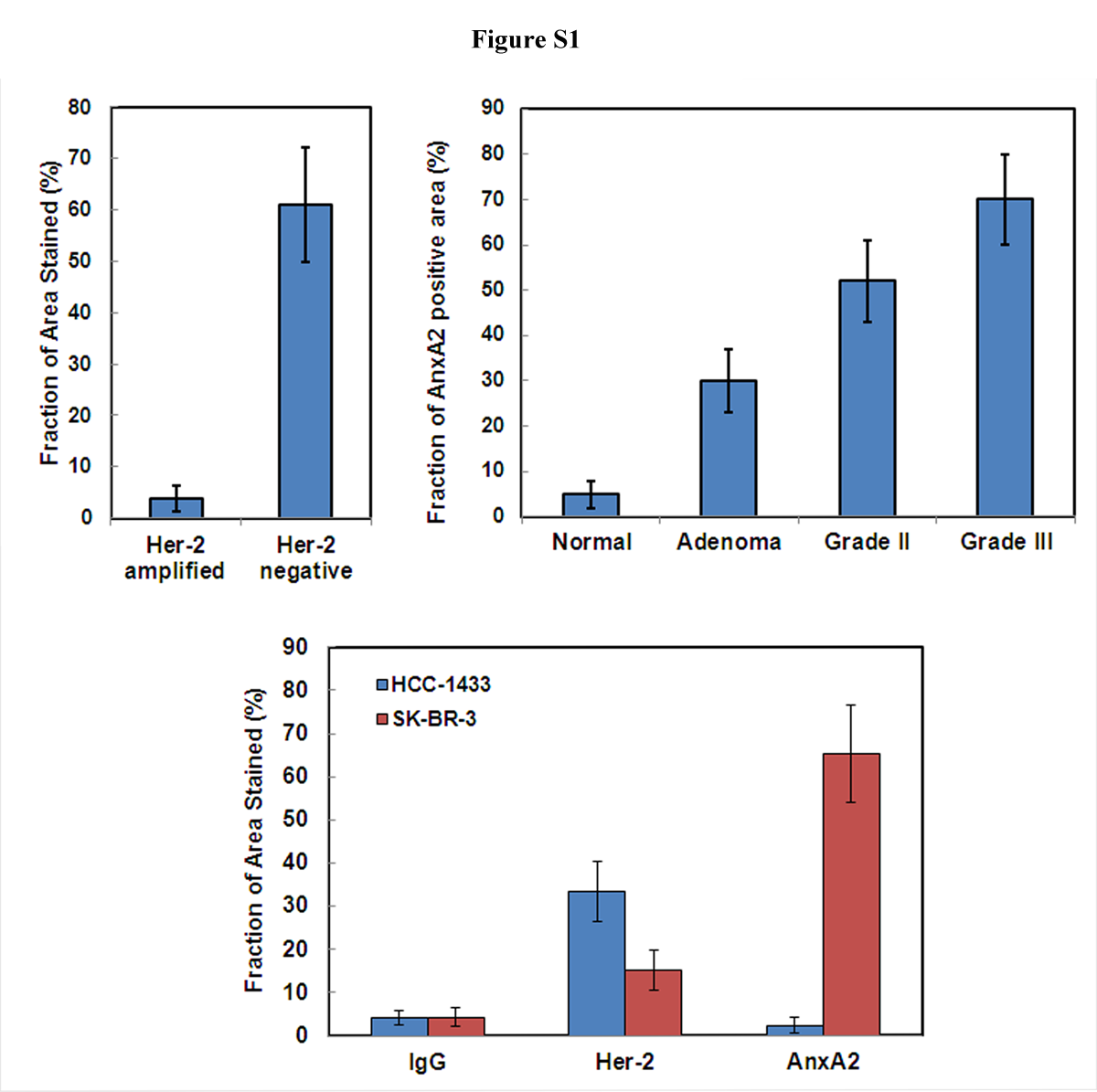

Supplement: Figure S1 — Inverse and positive correlation of AnxA2 expression in Her-2 negative cancer and their progression respectively. (A) Fraction of AnxA2 positive area in Her-2 amplified and Her-2 negative breast cancer. (B) Fraction of AnxA2 positive area in different stage of Her-2 negative breast cancer cases. (C) Fraction of AnxA2 positive area in breast cancer cells. Quantitative analysis of Figure 2Ci and 2Cii. (TIF) [file pone.0044299.s001.tif]

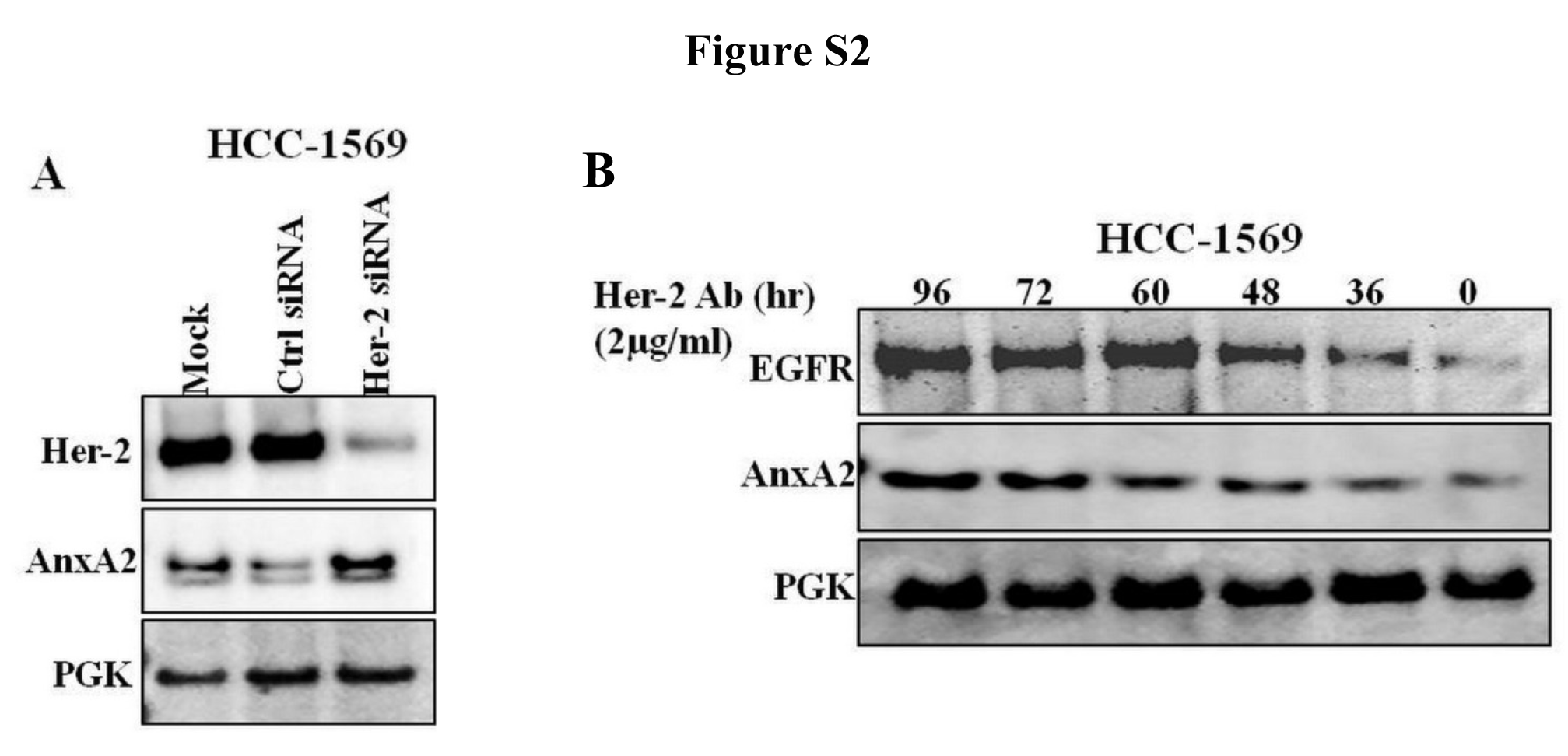

Supplement: Figure S2 — Her-2 downregulation in Her-2 amplified HCC-1569 cell line. (A) Her-2 amplified HCC-1569 cells were transfected with nonspecific siRNA or Her-2 siRNA. After 72 hours from transfection, cells were lysed and analyzed by Western blotting with Her-2 and AnxA2 antibodies. (B) The HCC-1569 cells were treated with Her-2 antibody (2 µg/ml) for different time intervals. After the respective treatment, cells were lysed and analyzed the expression of EGFR, AnxA2 and PGK by Western blotting. Blots shown are from one representative experiment and each experiment was repeated three times to ensure reproducibility normalized with EGFR. Similarly downstream signaling molecules like pERK1/2/ERK1/2, pP38/P38, pSTAT-3/STAT3 were analyzed using respective antibodies. (TIF) [file pone.0044299.s002.tif]
