# Supplementary material for: Reciprocal Regulation of Annexin A2 and EGFR with Her-2 in Her-2 Negative and Herceptin-Resistant Breast Cancer
Source: PLoS One. 2012 Sep 5;7(9):e44299. doi: 10.1371/journal.pone.0044299 (PMC3434131; doi:10.1371/journal.pone.0044299)
Supplement: Text S1 — Sequence of control, Her-2 and AnxA2 siRNA and AnxA2 shRNA. (DOC) [file pone.0044299.s003.doc]

**Supplementary Text S1**

**Materials:**

**RNA interference**

Small interfering RNA used this study were purchased from Dharmacon (Lafayette, CO). The pool of siRNA against AnxA2, Her-2 and non-targeted control were as below;

ON-TARGETplus SMARTpool L-010741-00-0005, Human AnxA2, NM_004039:

TARGET Sequence:

(1) CGACGAGGACUCUCUCAUU

(2) AUCCAAGUGUCGCUAUUUA

(3) AAAACCAGCUUGCGAAUAA

(4) GGAAGAAAGCUCUGGGACU

ON-TARGETplus SMARTpool L-003126-00-0005, Human ERBB2, NM_004448:

TARGET Sequence:

(1) UGGAAGAGAUCACAGGUUA

(2) GAGACCCGCUGAACAAUAC

(3) GGAGGAAUGCCGAGUACUG

(4) GCUCAUCGCUCACAACCAA

ON-TARGETplus Non-Targeting pool, D-001810-10-05

TARGET Sequence:

(1) UGGUUUACAUGUCGACUAA

(2) UGGUUUACAUGUUGUGUGA

(3) UGGUUUACAUGUUUUCUGA

(4) UGGUUUACAUGUUUUCCUA

**Sequence of shRNA**

**AnxA2 shRNA**

**Catalog No.** RHS4430-101067266

**Vector:** pGIPZ (lentiviral, sequence available at

<https://www.openbiosystems.com/Vector/VectorDetails.aspx?vn=pGIPZ>

**Hairpin sequence:**

TGCTGTTGACAGTGAGCGCCAGGAAAGAGGTTAAAGGAGATAGTGAAGCCACAGATGTA

TCTCCTTTAACCTCTTTCCTGATGCCTACTGCCTCGGA

**Mature Sense**

AGGAAAGAGGTTAAAGGAG

**Mature Antisense**

CTCCTTTAACCTCTTTCCT
